# Effects of Different Types of Human Disturbance on Total and Nitrogen-Transforming Bacteria in Haihe River

**DOI:** 10.3390/life12122081

**Published:** 2022-12-11

**Authors:** Peiyang Li, Tingyu Chen, Miao An, Ying Zhang, Yanying Li, Yang Li, Jing Wang

**Affiliations:** 1 Tianjin Key Laboratory of Animal and Plant Resistance, Tianjin Key Laboratory of Conservation and Utilization of Animal Diversity, Tianjin Normal University, Tianjin 300387, China; 2 Key Laboratory of Environmental Protection Technology on Water Transport, National Engineering Research Center of Port Hydraulic Construction Technology, Ministry of Transport, Tianjin Research Institute for Water Transport Engineering, Tianjin 300456, China

**Keywords:** Haihe River, human disturbance, microbial diversity, function potential, nitrogen cycle

## Abstract

Haihe River is the largest water system in North China and is injected into the Bohai Sea in Tianjin City. In this study, different types of human disturbance (urban sewage, industrial pollution, ship disturbance) were selected from the upper reaches of Haihe river Tianjin section down to the estuary that connected with Bohai Sea for evaluation. By metagenomic sequencing, the effects of different types of disturbances on bacteria communities in Haihe sediments were studied, with a special focus on the function of nitrogen-cycling bacteria that were further analyzed through KEGG comparison. By analyzing the physical and chemical characteristics of sediments, results showed that human disturbance caused a large amount of nitrogen input into Haihe River, and different types of human disturbance led to distinct spatial heterogeneity in different sections of Haihe River. The bacteria community was dominated by Proteobacteria, followed by Chloroflexi, Bacteroidetes, Actinobacteria and Acidobacteria. The relative abundance of each phylum varied at different sites as a response to different types of human disturbances. In nitrogen cycling, microorganisms including nitrogen fixation and removal were detected at each site, which indicated the active potential for nitrogen transformation in Haihe River. In addition, a large number of metabolic pathways relating to human diseases were also revealed in urban and pollution sites by function potential, which provided an important basis for the indicative role of urban river ecosystem for public health security. In summary, by evaluating both the ecological role and function potential of bacteria in Haihe River under different types of human disturbance, the knowledge of microorganisms for healthy and disturbed river ecosystems has been broadened, which is also informative for further river management and bioremediation.

## 1. Introduction

Rivers are one of the core parts of aquatic ecosystems and are carrying significant ecological and economic functions for urban construction and human activity. In contrast to natural rivers, urban rivers are not only improving the ecological environment of cities, but also play important roles in water supply, irrigation and production [1]. Under rapid urbanization, industrial wastewater and sewage, pesticide and fertilizer residue were introduced into rivers along with surface runoff [2,3]. As a consequence, the concentrations of organic matter, nutrient elements, inorganic pollutants and, especially, the biologically available active nitrogen increased dramatically [4,5]. However, the sediment that tends to concentrate more pollutants than the water body and collect at the bottom of rivers can become a new source of pollution to the overlying water, leading to continued eutrophication of rivers [6,7,8]. River sediments provided sites for microbial growth and reproduction, which further facilitate the material exchange in rivers [9]. Therefore, sediments are ideal targets for exploring the biogeochemical cycling of matter and the structure of microbial communities under different anthropogenic contaminants.

Microorganisms in rivers link in the aquatic food chain [10] and play an important role in energy flow and biogeochemical cycling of elements [11,12], so as to maintain water self-purification and balance the payments in river ecosystems [13]. Nitrification and denitrification are catalyzed by relating nitrogen-transforming genes, including *amo*, *nir*, *nar*, *nor*, etc., which can convert or remove nitrogen from water as a way of water self-purification [14,15]. As a fact, the community structure of microorganisms can be altered under various anthropogenic pollution [16,17,18,19]. This shows that microorganisms are sensitive to changes in their environment and highly susceptible to environmental changes [20]. Therefore, it is important to understand the community structure and spatial distribution of microorganisms in river ecosystems under anthropogenic disturbance.

The Haihe basin is one of the seven major water systems in China, and is the largest water system in northern China, with a length of 76 km and an average water depth of 6–9 m. The tributaries of the Haihe basin eventually form the main stream of the Haihe River in Tianjin city, but with the increasing population density and the continuous development of industry and agriculture, The water quality in the upper reaches of the Haihe River reached inferior category V between years of 1998–2013 [21], posing a serious threat to residents, animals and plants in the watershed. Moreover, the diversity, distribution and community structure of bacterial communities in river ecosystems under different types of anthropogenic disturbances have been rarely reported, leaving a blank for such information in Haihe River. The application of shotgun metagenomic sequencing with phylum level in taxonomic diversity and functional adaptability analysis of bacteria communities have been reported by several studies [22,23,24,25]. Although a large number of studies have employed amplicon sequencing targeting the conserved 16S rDNA, regarding their advantages in identifying bacterial populations, their limited resolution and inability to infer the functional capacity of the microbial communities are major limitations [26,27]. Shotgun metagenomic sequencing provides a better understanding of the structure and diversity of microbial communities as well as their metabolic potential [26], by detecting microbial changes in river ecosystems under human disturbance, especially the response of the functional microbes to different degree of pollutants, poses important theoretical value and practical significance for the evaluation of healthy rivers and the microbial ecological restoration of polluted rivers.

In this study, five sites with representative human disturbance characteristics along Haihe River from the Tianjin section were selected. The distinct contamination inputs at each site were identified. By way of metagenomic sequencing, combined with a functional prediction by KEGG, the anthropogenic impact on bacteria communities in the river sediment were studied. The main objectives of this study were (1) to evaluate the community composition of total bacteria in the Haihe River sediments, (2) to clarify the effects of environmental factors on bacterial communities, and (3) to reveal the response of the functional group, especially the nitrogen-transforming bacteria under different types of human disturbance in river ecosystem. Results from this study would improve the recognition of an important role of microbes in urban rivers, and provide evidence for domestic microbial remediation for river management in the future.

## 2. Materials and Methods

### 2.1. Sample Collection

**Figure 1:**
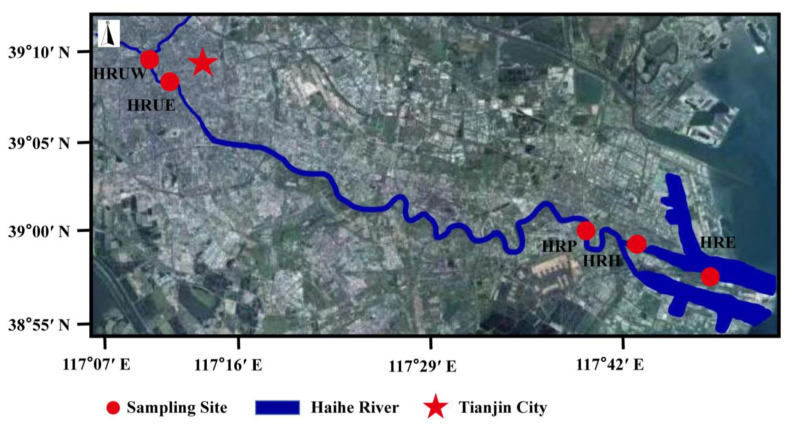
Map of sampling sites in the Haihe River.

In this study, five sites from Haihe River across Tianjin city and the Binhai New Area were selected based on the different types of human disturbance (Figure 1). From the upper stream near Tianjin West and East Railway Station, two sites with urban sewage and anthropogenic activity impact were selected as HRUE and HRUW; in the lower reaches, the HRP site was selected to reflect the impact of industrial pollution, where the adjacent chemical plant discharged for the last several years; down to the estuary, two sites of HRH and HRE were selected to represent the ship manufacture and shipping disturbance impact on the river. Detailed physicochemical characteristics of each site was displayed in Table 1.

Both surface water and sediments were sampled at triplicate. The sediment samples were collected by stainless steel 0.1 m^2^ GyO’Hara box corer at a depth of 10 cm, and the top 2 cm samples were removed before subsampling to avoid disturbance during sampling. After homogeneity of three sampling cores, three subsamples (60 g each) from each site were collected randomly. The water samples in the bottle were transferred to the laboratory on ice, and the sediment samples in sterile ziplock bags were attached to dry ice during transportation before being stored in a refrigerator at −80 °C for DNA extraction.

### 2.2. Environmental Parameters Analysis

Samples were collected and physical and chemical parameters were determined either in situ or without disturbance after being transported to the laboratory. At each site, the salinity, dissolved oxygen (DO), pH and temperature of seawater was recorded in situ by employing the Lovibond Tintometer (Tintometer, Amesbury, UK) handheld multiparameter instrument (model:SD335). Sediment samples were collected 3 times at each station with a mud sampler, and 3 portions (60 g) were randomly selected after fully mixing the subsurface samples. The physicochemical parameters of the sediments, including NH_4_^+^-N, NO_3_^−^-N, NO_2_^−^-N, S, P, Fe and total nitrogen content, were measured using procedures as previously reported [28].

### 2.3. Extraction of Total Community DNA and Sequencing

Three random subsamples were collected from five sites, a total of fifteen samples were applied for metagenomic analysis. DNA from sediment was extracted and cleaned as previously reported [29]. Briefly, DNA was extracted using a DNeasy Power Soil Pro Kit (QIAGEN, Germantown, MD, USA) and concentrated using a DNA Clean & Concentrator kit (Zymo, Irvine, CA, USA). Using a Qubit 2.0 fluorometer from Life Technologies (Budapest, Hungary), clean and concentrated DNA was measured and kept at −20 °C for storage. DNA for shotgun metagenomics was sequenced using an Illumina HiSeq 6000 instrument with paired-end 300 bp reads (Shanghai Origingene Bio-pharm Technology Co.Ltd., Shanghai, China) [30].

### 2.4. Taxonomic and Functional Analysis of Metagenomes

In total, fifteen samples were treated independently for metagenomic analysis. Covaris M220 was used to fragment the DNA sequence, and then clean reads sequence was obtained by fast software quality control. To improve the quality and reliability of the subsequent analysis, the original sequencing data was cleaned as follows. Firstly, bases with a mass value below 20 at the tail of reads were filtered, a window of 50 bp was set, and the window was moved from the head. If the average mass value in the window was lower than 50, the back-end bases were cut off from the window. In addition, reads containing the number of N bases >2, reads containing adaptor joint contamination, and the reads below 50 bp were removed. If the sample was derived from a host whose genome had been published, reads were aligned with the host DNA sequence by software BWA, and contaminated reads with high similarity were removed. Megahit software was used to splice the sequence fragments, and K-mers were iterated from small to large to achieve fewer gaps and long contigs. Statistics of assembly results are presented in Table 2. MetaGeneMark software v.3.38 was used to predict the gene of contig in the splicing results. The fragments with length ≥ 100 bp were selected and translated into amino acid sequences. DNA was clustered by CD-HIT software (95% identity, 90% coverage). The BLASTP software was used to compare the clustering gene set with the NR database, and the taxonomic information in the NR database was used to obtain the annotation results of species. Genes were compared to the KEGG database in order to identify potential metabolic pathways [31]. If the similarity search produced an expectation E value less than 1 × 10^−5^, it was considered a match. The statistical analysis of all KO (KEGG Orthology) numbers versus KEGG route functional hierarchy.

### 2.5. Statistical Analysis

Origin 2019 and IBM SPSS Statistic 23 softwares were used to analyze the data. Pearson correlation analysis and linear regression analysis were performed using IBM SPSS Statistic 23 to detect the correlation between physical and chemical parameters and microbial composition. The principal component analysis (PCA) was calculated by R 3.6.3 with s vegan package to evaluate the spatial distribution of the bacteria community. The typical correspondence analysis was applied to analyze the bacterial community dynamics with eight environmental variables (see Table 1).

### 2.6. Data Deposition

The sequences were deposited in NCBI under accession numbers SRR22252632-SRR22252646.

## 3. Results

### 3.1. Subsection Physicochemical Characteristics of Haihe River

The physicochemical characteristicsof sediments from Haihe River were displayed in Table 1. Based on the diverging difference in salinity, three ecological zones were distinguished from the five sites, which were the urban zone (HRUE and HRUW, salinity < 1), pollution zone (HRP, salinity = 33.37) and estuary zone (HRH and HRE, salinity > 20). All the sediments were in alkaline condition (pH values ranged between 7.28 to 8.42), with the highest pH value occurring in the pollution site. Both salinity and pH together with DO show the lowest value in the urban zone and the highest value in the pollution zone. As a fact, the nutrients were accumulated in urban zone, with the peak values of TP, TN, NH_4_^+^-N, NO_3_^−^-N and NO_2_^−^-N observed in sites HRUE and HRUW. Nitrogen in sediments exists mainly in the form of NH_4_^+^-N (Table 1). The values of these parameters (except nitrate nitrogen) were reliably associated with the concentration of TP (*p* < 0.05) (Table 3).

### 3.2. Microbial Community Structure of Sediment Samples

**Figure 2:**
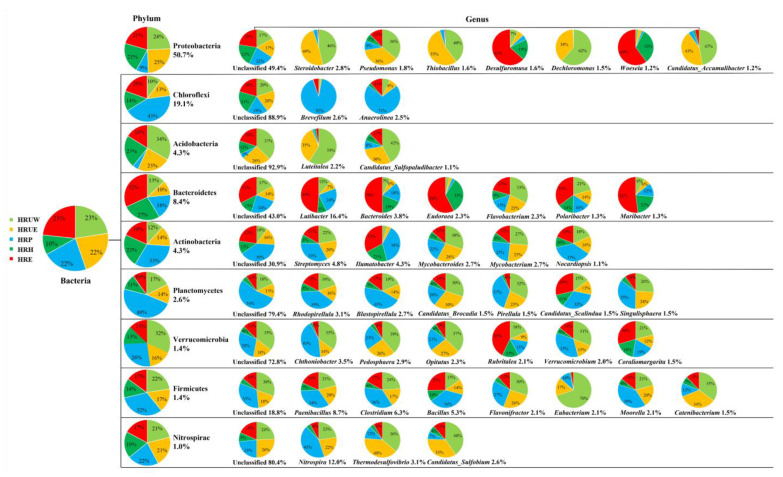
Community composition of bacteria at each site represented with phylum and genus level. The percentage after phylum name was the average relative abundance of the 3 replicates from each sampling site. At genus level, only the relative abundance that greater than 1% was shown in the figure.

In total, fifteen sediment samples from five sites with three replicates were sequenced. The structures and abundance of bacterial communities in sediment samples from different sites were profiled in Figure 2. At phylum level, the mostly detected bacteria were Proteobacteria (50.7–61.1%), Chloroflexi (10.9–43%), Bacteroidetes (2.7–8.4%), Acidobacteria (4.3–9.0%), Actinobacteria (3.0–5.3%), etc., with different relative compositional proportion at each site. While at genus level, the unclassified genera within each phylum reached up to 92.9%. Although a large amount of diverse genus was revealed at each site, the low relative abundance (mostly < 1%) restricted their potential function prediction in the following procedures. Proteobacteria reached 58.6–61.2% in the Urban West site, but only 21.3–22.1% in the pollution site, as shown in Figure S1. Phylum of Proteobacteria is mainly composed of classes of Gammaproteobacteria, Deltaproteobacteria, Betaproteobacteria and Alphaproteobacteria. Among which, Gammaproteobacteria was the most dominant class (17.6%), and the dominant genus was *Steroidobacter*, with an abundance of 2.8% among all the genera within Proteobacteria (Figure 2). In the pollution site, the relative abundance of Chloroflexi was as high as 40.1–41.4%, but only 13–20% at the other four sites (Figure S1), within which, the composition of genera *Brevefilum* and *Anaerolineae* was significantly higher than those in the other 4 sites (*p* < 0.01) as well, and *Dehalococcoidia*, *Chloroflexia*, *Candidatus_Thermofonsia* were also high in composition in HRP (*p* < 0.05). Genera *Woeseia* and *Desulfuromusa* from Proteobacteria, *Lutibacter*, *Bacteroides*, *Eudoraea*, *Polaribuster* and *Mariactor* from the Bacteroidetes phylum were predominantly distributed in the HRE site (>50%) (Figure 2). It was worth mentioning that phylum Nitrospirae was evenly detected at each site with a relative abundance of 1%, and the genera of *Nitrospira*, *Thermodesulfovibri*o and *Candidatus_Sulfobium* were dominated at the both urban sites.

**Figure 3:**
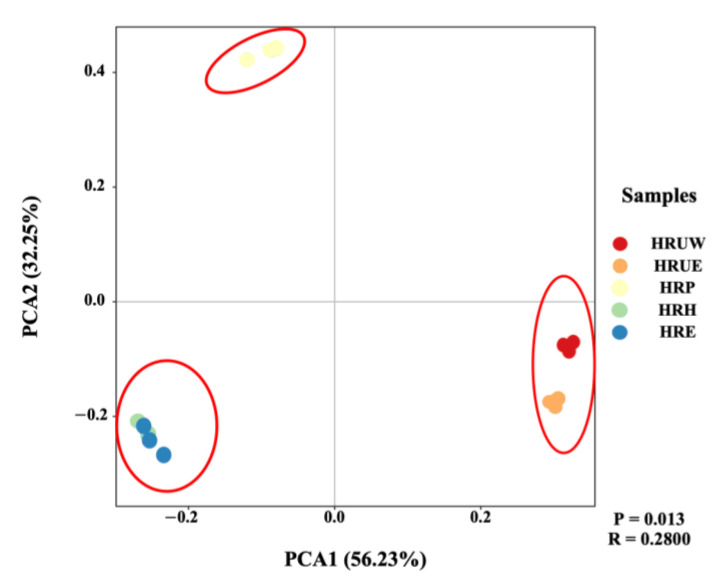
PCA analysis of bacterial communities based on metagenome.

PCA was further performed to reveal the distribution pattern (Beta diversity) of microbial communities in Haihe River from each site (Figure 3). The explanation degrees of the first principal component (PC1) and the second principal component (PC2) in the PCA ranking diagram of microbial community were 56.23% and 32.25%, respectively. All the samples were divided into three clusters, which was in accordance with the three typical ecological zones (Figure 3). Samples from the HRUE and HRUW were grouped together; samples from HRH and HRE were also closely clustered, while the three zones were separated distinctively, showing the big heterogeneity of bacterial community structure in the studied sites. The distance between the pollution zone and the other two zones indicated a considerable variation in bacterial composition. Although the pollution zone was located close to the urban area and suffered high anthropogenic impact, the bacterial community were differentiated.

### 3.3. Correlations between Environmental Parameters and Bacterial Community

**Figure 4:**
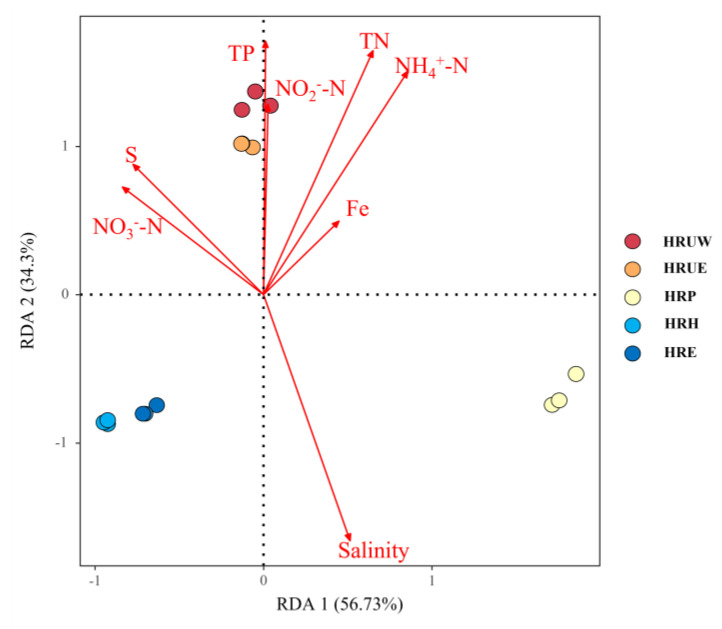
RDA analysis of environmental factors and sediment samples from the Haihe River.

Redundancy analysis (RDA) was applied to reveal the correlations between the physicochemical parameters of sediment and bacterial communities in sediments (Figure 4). The first two axes explained up to 57.12% of the total variation in bacterial community structure for RDA 1 and 34.54% for RDA 2, which indicated that the selected sediment physicochemical factors could explain the variations of community composition in sediment. The results show that salinity, TP, TN, NH_4_^+^-N, NO_2_^−^-N (*p* < 0.01) and S (*p* < 0.05) were the key impact factors shaping the community composition. Among which, TN, NH_4_^+^-N, NO_3_^−^-N, NO_2_^−^-N, S and TP were positively correlated with the samples from HRUW and HRUE, indicating the microbial communities from these sites were significantly influenced by multiple parameters. While salinity was positively correlated with HRP, HRH and HRE, but negatively correlated with HRUW and HRUE. TN contents in Haihe River sediments were significantly high, and TN together with NH_4_^+^-N were positively correlated with bacterial community structure in significance (*p* < 0.01).

**Figure 5:**
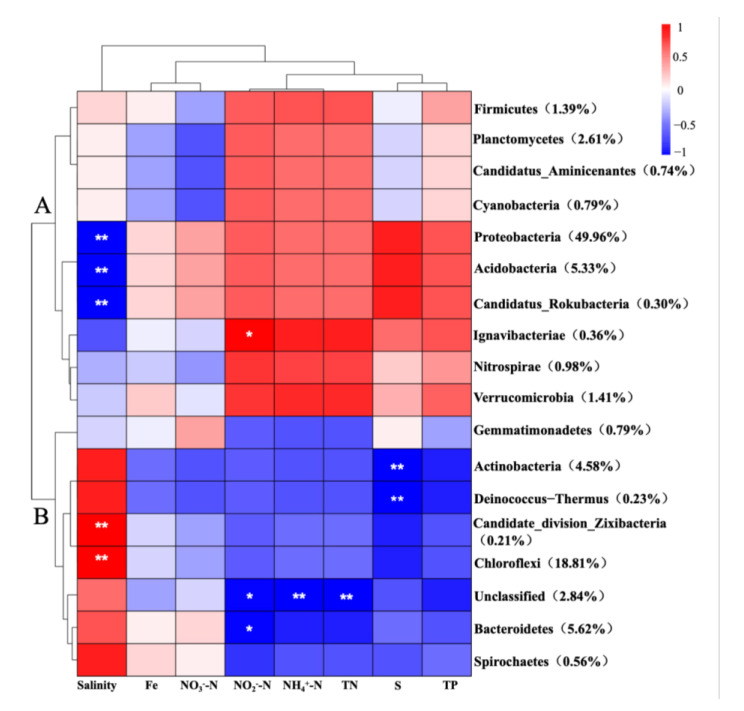
Spearman correlation heat map analysis of microorganisms in Haihe river sediments. (* represents the correlation significance between microorganisms and environmental parameters. * *p* < 0.05, ** *p* < 0.01).

Further, a heatmap and cluster analysis were conducted to verify the correlation significance between environmental parameters and the dominant bacteria phylum (Figure 5). Based on the results from the cluster, the top 18 dominant phyla were divided into two groups. Clade A was mainly composed of Proteobacteria, Acidobacteria, Verrucomicrobia and Planctomycetes, while Clade B bacteria were constructed mainly by Chloroflexi, Bacteroidetes and Actinobacteria. Among the eight variables, salinity acted a key role in determining the bacterial diversity and was positively correlated with the composition of Actinobacteria, Deinococcus-Thermus, Candidate_division_Zixibacteria, Chloroflexi, Bacteroidetes and Spirochaetes (*p* < 0.01), but was negatively correlated with Proteobacteria, Acidobacteriais, Candidatus_Rokubacteria (*p* < 0.01). TN, NH_4_^+^-N and NO_2_^−^-N were negatively correlated with Clade B bacteria and especially inhibiting the growth of Bacteroidetes and Unclassified bacteria, but were positively correlated Clade A bacteria, such as Verrucomicrobia, Nitrospirae and Ignavibacteriae. Sulphur was negatively correlated with Clade B bacteria but was positively correlated with Proteobacteria, Acidobacteriais and Candidatus_Rokubacteria. TP promoted the relative abundance of Clade A bacteria significantly, but was observably negatively correlated with Clade B bacteria. These results revealed that the bacterial community were exquisitely sensitive to nutrient parameters, including TN, NH_4_^+^-N, NO_2_^−^-N, TP and S.

### 3.4. Function Potential of Total and Nitrogen-Transforming Bacteria

**Figure 6:**
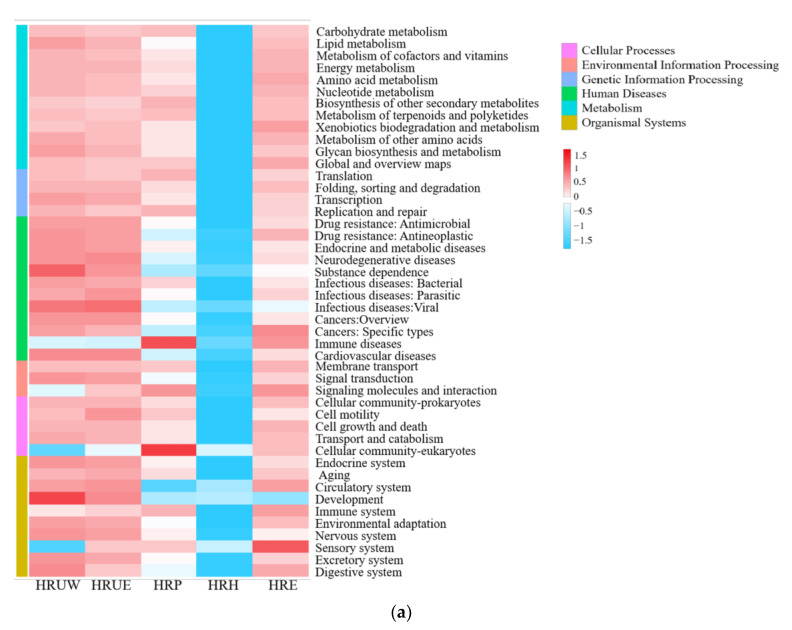

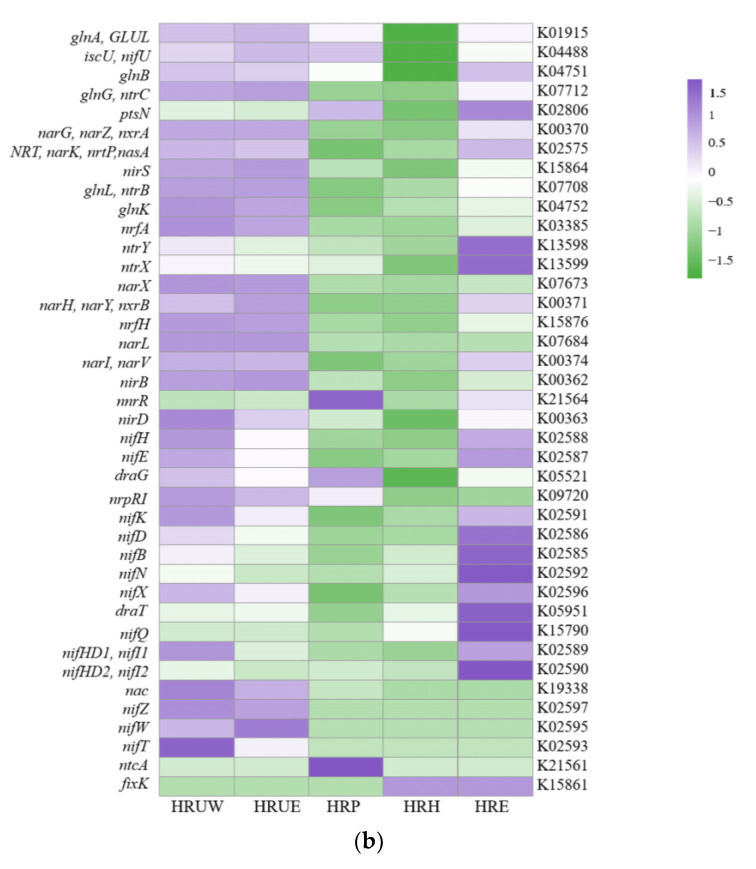
KEGG comparison analysis of (**a**): bacterial functional classes and (**b**): nitrogen cycle-related KO prediction heat map.

The results of macro genome sequencing were compared with the KEGG database and six categories of bio-metabolic pathways were identified: metabolism, human diseases, organismal systems, cellular processes, genetic information processing and environmental information processing (Figure 6a). With the aim to obtain a deeper understanding of the nitrogen cycle in sediments that drove by microorganisms, functional genes involved in nitrogen input (nitrogen fixation) and nitrogen export (denitrification) were further annotated by the KEGG ortholog group (KO) database. A correlation of the KOs associated with nitrogen cycles and their abundances revealed that genes associated with nitrogen fixation (Figure 6b), such as K01915 (*glnA*) and K04751 (*glnB*) were abundant at all sites, however, genes of K02586 (*nif D*), K02585 (*nif B*), K02592 (*nif N*), K02596 (*nif X*), K15790 (*nif Q*), K02589 (*nifHD*1, *nifI1*) and K02590 (*nifHD2*, *nifI2*) were mainly distributed in the HRE, indicating a strong nitrogen fixation capacity in the Haihe River basin. The genes associated with the typical denitrification pathways, including K00370 (*narG*), K02575 (*narK*), K03385 (*nrfA*), K07673 (*narX*) and K07684 (*narL*) were abundant in sites from urban zone and higher than that in the other two zones. It was noteworthy that 12 pathways associated with human diseases are distributed in each occupancy, those bacteria that related to human diseases were lurking in the Haihe River and should be given concern in priority.

## 4. Discussion

Rivers are suffering different kinds of anthropogenic disturbances and the original ecological balance are destroyed with the developing civilization and industrialization [32]. Anthropogenic disturbance, which mainly includes sewage, industrial wastewater and ship disturbance in Haihe River, have increased due to a lack of management since the 1970s [33]. As a fact, different anthropogenic disturbances have caused typical spatial heterogeneity within the studied sites. The discharge of domestic sewage and the dense population caused high concentrations of NH_4_^+^-N and TP in sites HRUE and HRUW from urban areas. The heavy pollution of TN in the two sites from urban reaches 209.3–266.7 g/kg, which indicates that the Haihe river is suffering from NH_4_^+^-N accumulation in a long time run [34]. Rivers that are adjacent to urban areas act as a major sink for discharged wastewater, which may contain amounts of organic pollutants and excessive nutrients, altering the environmental characteristics of river water and sediments [32,35], such as a decrease the dissolved oxygen, destroy aquatic ecosystems and result in eutrophication [36]. The zone of pollution is specialized by industrial wastewater discharge from the chemical plants in up streams. Wastewater generated during the production of fine chemicals contains a high concentration of organic matter, salt, ammonia nitrogen, resulting in a significant increase in sediment salinity and DO. In contrast to other anthropogenic sources of pollution, industrial areas (HRH, HRE) are located at the mouths of the sea rivers and have high salinity and NO_3_^−^-N concentrations, which have been found in previous studies to carry harmful aquatic organisms and pathogens during the transport of goods and discharge of ballast water, causing pollution of the river environment [37]. Overall, various environmental factors drive the development of a diversity of microbial community systems to cope with different sources of pollutants.

The bacteria community is an important component in urban river ecosystems and a key driver for energy flow and nutrient cycling in urban rivers [12]. Large amounts of organic pollutants and excess nutrients can alter the characteristics of river sediments and the structure of bacterial communities [32,38]. The main bacterial phyla in this study include Proteobacteria, Chloroflexi, Bacteroidetes, Acidobacteria and Planctomycetes, which are also dominant populations in the sediments of the eutrophic Nanfei River, the North Canal [39], the Santa Ana River in Southern California [40], and are basically consistent with the bacterial composition of river sediments polluted by heavy metals, organic pollution and papermaking wastewater [41]. The phylum of Proteobacteria is dominant in urban areas (HRUE, HRUW) and contains many nitrogen-fixing bacteria that degrade organic matter while performing systematic nitrogen and phosphorus removal functions [42], which may also be driven by high nutrient sources from municipal wastewater (Table 1 and Figure 2). This observation is similar to previous reports that the abundance of Proteobacteria is significantly correlated with nutrient content [38]. Within phylum of Proteobacteria, Alphaproteobacteria is a typical dominant group in freshwater bacterial communities, including nitrogen-fixing bacteria that can coexist with plants and provide greater nitrogen fixation in rivers [43]. Betaproteobacteria is more likely to survive in a polluted environment and can be used as an ecological indicator for environmental quality monitoring and evaluation [44]. Studies have shown that Gammaproteobacteria are widely distributed in eutrophic environments and is modulated by pH and nutrient availability [45,46]. In this study, the high abundance of Gammaproteobacteria in the sediments of the urban area is possibly due to high nitrogen content, while the sharp decrease in Gammaproteobacteria abundance in the polluted area can be modulated by high pH and low nutrient availability.

It is worth noting that Chloroflexi, as the dominant phylum in the zone of pollution site (HRP), may be associated with chemical plant effluent discharge. Previous studies have shown a significant increase in Chloroflexi abundance in intertidal areas, pharmaceutical wastewater sludge and contaminated river sediments [47,48], in where they are involved in the degradation of organic pollutants and act as an indicator microorganism for pollution [49]. This clue also, in part, explains the relative low nitrogen content in the pollution area of Haihe river (Table 1).

Acidobacteria are common and abundant members of the bacterial community in freshwater sediments, and their relative abundance is high in urban areas. Studies have suggested that the abundance of Acidobacteria is significantly correlated with pH, and Acidobacteria prefers an environment with low pH of approximately 5.5 [50,51]. The pH of sediments in the pollution zone is significantly higher than that in the other area (Table 1), which possibly accounts for the lower abundance of Acidobacteria in this site (Figure 2). In another hand, Acidobacteria also contributes to metabolism in sediments under acidic conditions [52], thus promoting the activation of steady-state heavy metals in sediments and pose a secondary pollution threat to the aquatic environment. Percent of community abundance in phylum level indicates that the distribution of Acidobacteria subdivisions in river sediment varies with trophic status [53], the trophic variations in the five studied sites also in help with understanding the distribution of Acidobacteria subdivisions in the Haihe river sediments. In turn, their metabolism in function also affects the trophic status of the river.

Bacteroidota, another major phylum in sediments, are also highly environmental-adapted bacteria [54]. Some species in this phylum have been found to be effective alternative fecal indicators [55] and studies have found that Bacteroidota are associated with the human gut microbiota [56], which may be associated with the discharge of untreated domestic sewage into rivers. Firmicutes can participate in the degradation and transformation of multiple substances and elements, which is then dominant in lightly eutrophic sites (HRUW, HRUE, HRH and HRE). Planctomycetes are widely distributed in natural and artificial ecosystems, such as seawater, freshwater, soil and wastewater [57,58,59,60]. Planctomycetes play an important role in biological nitrogen removal either from the nature environment or in high ammonia wastewater [61]. Salinity [62] and the ratio of NH_4_^+^/NO_2_^−^ [63] are the key factors affecting the Planctomycetes community differentiation related to the anammox process. In pollution areas, the increase in Planctomycetes relative abundance indicates that the anammox process may be intensified and, consequently, help to explain the lower nitrogen content in the site HRP.

Studies have indicated that salinity and nitrogen are the major factors relating to microbial communities [17,18,19]. Our study also confirms this result. Excess TN in rivers is mainly in the form of NH_4_^+^-N, which is an important nitrogen source for heterotrophic bacteria. Alphaproteobacteria, Betaproteobacteria and Gammaproteobacteria contain a great number of denitrifying bacteria, which are capable of autotrophic or heterotrophic denitrification, and play important roles in nitrogen, phosphorus, sulfur and organic matter cycling in sediments. Nitrospirae, an important nitrite-oxidizing bacteria that can oxidize nitrite to nitrate [64], which may be responsible for the high nitrate in the industrial zone. As the important classes for nutrient release in sediments, Proteobacteria and Nitrospirae are also early warnings for the occurrence of algal bloom. The predominance of these two groups in the sediments of Haihe river is also a good indicator of the deterioration of water quality. Nitrogen levels are also key environmental factors affecting the functional structure of bacterial communities in river sediments. Therefore, an intensive study of the genetic function of nitrogen metabolism in bacterial communities from polluted urban river sediments is necessary.

Various studies have applied metagenomes to explore environmentally relevant genes and annotated the function with KEGG [65,66]. Previous studies have shown that rivers and lakes are important sites for the removal of imported nitrogen from the terrestrial environment [67,68,69]. The abundance of denitrification genes is significantly related to water quality and sediment properties [70]. In this study, we observed a high abundance of *nirS* in urban areas, which was a key gene in nitrite reduction and nitric oxide production [29], as well as a major influencer of denitrification potential in denitrifying microbial communities [48]. Denitrification potential increased with the expression of *nirS* gene in soil [71]. The abundance and distribution of pairs of functional genes can also be influenced by different pollution sources (Figure 6b). For example, the relative abundance of *narG* gene was found to be higher in domestic pollution than in agricultural and industrial pollution in another urban river sediment [72], which is similar to the results observed in this study. The high abundance of *nrfA* gene was argued to be related to water eutrophication, which may also be a reason for the high NH_4_^+^-N content in water bodies. The abundance of *nrfA* in the sediments of Chaohu Lake was found to play an important role in Cyanobacteria blooms [73]. In contrast to denitrification, many microbial communities in sediments also have a high relative abundance of nitrogen fixation genes, such as high expression of *nifD*, *nifH*, *nifN*, which can help us to understand the nitrogen fixation capacity of microorganisms in riverine ecosystems [74]. The high abundance of *nifH* in agricultural pollution than in other sources of pollution may be related to the use of organic fertilizers, which might promote the abundance of related genes [65]. The above results suggested that bacterial communities in sediments have strong functional potential in anthropogenically disturbed rivers.

Intensive industrial activities and increasing urbanization are associated with the continued emergence of antibiotics [75]. The widespread use of antibiotics can lead to increased resistance to microorganisms in the environment [76]. Resistance genes are distributed in various environmental media and may enter the human body directly or indirectly [77]. In this study, inappropriate discharges of effluents from hospitals and chemical plants causing antimicrobial resistance (ARGs) were found to have a high abundance in urban areas. A high abundance of ARGs was detected in tributaries of the Yangtze River Delta estuary with a high potential for horizontal gene transfer (HGT) [78,79], which would threaten the health of the surrounding population. Moreover, a large number of pathogenic Gammaproteobacteria such as *Salmonella*, *Legionella* and *Vibrio* can spread with different media and also pose a significant risk to public health [80]. Nowadays, with the impact of the epidemic, we should pay more attention to the environment and the spread of human diseases, and to the treatment of water bodies and sediments in the region.

## 5. Conclusions

In this study, bacterial community structure from an urban river sediment was investigated by using metagenome sequencing technology. From three typical disturbances to Haihe River, different bacterial communities were identified in the urban zone, pollution zone and industry zone with respective distinguishable physicochemical characteristics. Compared to the other two zones, changes in the composition of the bacterial community were tremendous in the pollution zone, which was determined by multiple local environmental parameters. Functional potential from the bacteria displayed variations of nitrogen-transforming genes in the studied sites, which might contribute to the cycling of nitrogen in the sediment of Haihe River. Moreover, the discovery of human disease-related pathways displayed an important potential of public health security posed by microorganisms from river ecosystems. This study provides theoretical support for the restoration of urban watersheds. Based on the composition of bacterial community and their relationship with environmental factors, regular monitoring and remediation strategies should be conducted to prevent further deterioration of river water quality.

## Figures and Tables

**Table 1:** Physicochemical properties of sediment samples.

Sample Name	Sampling Site	Location	Temp (°C)	Salinity (mg/kg)	DO (mg/L)	pH	NO_2^−^_-N (mg/kg)	NO_3^−^_-N (mg/kg)	NH_4^+^_-N (mg/kg)	TN (mg/kg)	TP (mg/kg)	S (mg/kg)
HRUW	Urban West	117.17 E39.1567 N	11.00	1.67 × 10^3^ ± 0.03 e	8.70	7.42	0.41 ± 0.04 a	6.62 ± 0.05 c	209.30 ± 3.68 b	3.42 × 10^3^ ± 24.49 a	1.66 × 10^3^ ± 28.28 a	5.07 ± 0.04 c
HRUE	Urban East	117.19 E 39.1308 N	9.50	5.04 × 10^3^ ± 0.60 d	8.11	7.28	0.17 ± 0.01 b	7.86 ± 0.07 b	266.70 ± 0.94 a	3.37 × 10^3^ ± 327.85 a	1.09 × 10^3^ ± 17.00 b	6.52 ± 0.07 a
HRP	Pollution	117.67 E 38.9975 N	10.20	3.34 × 10^4^ ± 1.47 a	18.95	8.42	0.17 ± 0.00 b	1.19 ± 0.04 e	165.00 ± 3.56 c	2.14 × 10^3^ ± 91.77 b	4.7 × 10^2^ ± 3.09 d	4.04 ± 0.04 e
HRH	Harbor	117.72 E 38.9896 N	10.10	2.07 × 10^4^ ± 0.76 c	9.83	7.57	0.15 ± 0.00 b	1.84 ± 0.05 d	6.90 ± 0.22 e	5.50 × 10^2^ ± 12.39 c	2.6 × 10^2^ ± 6.53 e	4.38 ± 0.05 d
HRE	Estuary	117.81 E 38.9605 N	10.40	3.13 × 10^4^ ± 0.48 b	9.78	7.79	0.15 ± 0.00 b	9.80 ± 0.10 a	23.30 ± 0.34 d	1.10 × 10^3^ ± 28.67 d	5.9 × 10^2^ ± 5.73 c	5.86 ± 0.10 b

Different lowercase letters in the figure stand for significant differences at *p* < 0.01 level.

**Table 2:** Statistics of assembly result.

Sample ID	NO. of Contigs	Total Len	N50	N90	Average Len	Max Len
HRUW1	372,582	297,816,176	760	532	799.3	19,500
HRUW2	368,534	290,566,213	746	531	788.4	35,316
HRUW3	367,011	283,507,046	732	530	772.5	30,185
HRUE1	364,746	285,809,465	740	529	783.6	31,584
HRUE2	321,303	257,675,979	758	531	802.0	46,219
HRUE3	301,958	240,403,741	752	531	796.2	45,373
HRP1	500,496	476,635,987	947	547	952.3	52,099
HRP2	466,734	433,600,392	918	545	929.0	38,967
HRP3	452,588	420,081,579	918	545	928.2	38,967
HRH1	371,499	314,017,265	800	535	845.3	47,673
HRH2	426,832	371,053,744	828	537	869.3	65,887
HRH3	466,448	405,576,702	829	538	869.5	89,681
HRE1	464,423	402,207,176	825	538	866.0	39,033
HRE2	448,975	383,701,166	813	537	854.6	39,271
HRE3	405,101	343,081,767	804	536	846.9	30,827

Each Contigs sequence is sorted according to its length, and the length value of each contigs is scanned one by one from the largest to the smallest for accumulation. When the accumulation value exceeds 50% of the total length of all contigs for the first time, the length value of the scanned sequence is N50. Compared with the average length of the sequence, N50 can more accurately represent the effect of the sequence stitching. Max len specifies the sequence length of the longest contig.

**Table 3:** Correlation analysis between physical and chemical parameters of sediments.

	Salinity	TP	S	TN	NH_4_^+^-N	NO_3_^−^-N	NO_2_^−^-N
Salinity	1	−0.68 **	−0.46	−0.58 *	−0.48	−0.24	−0.59 *
TP		1	0.59 *	0.84 **	0.78 **	0.48	0.74 **
S			1	0.37	0.48	0.88 **	0.10
TN				1	0.91 **	0.12	0.82 **
NH_4_^+^-N					1	0.19	0.72 **
NO_3_^−^-N						1	−0.15
NO_2_^−^-N							1

The degree of significance of Spearman Rank correlations is marked by the stars. * (*p* < 0.05, significant correlation), ** (*p* < 0.01, highly significant correlation).

## Supplementary Materials

The following supporting information can be downloaded at: https://www.mdpi.com/article/10.3390/life12122081/s1, Figure S1: Composition of bacteria communities in 15 sediment samples with relative abundance at phylum level.
