# Supplementary material for: Effects of Different Types of Human Disturbance on Total and Nitrogen-Transforming Bacteria in Haihe River
Source: Life (Basel). 2022 Dec 11;12(12):2081. doi: 10.3390/life12122081 (PMC9781767; doi:10.3390/life12122081)
Supplement: Supplementary file 1 [file life-12-02081-s001.zip › life-2000355-supplementary.pdf]

Supplementary material:

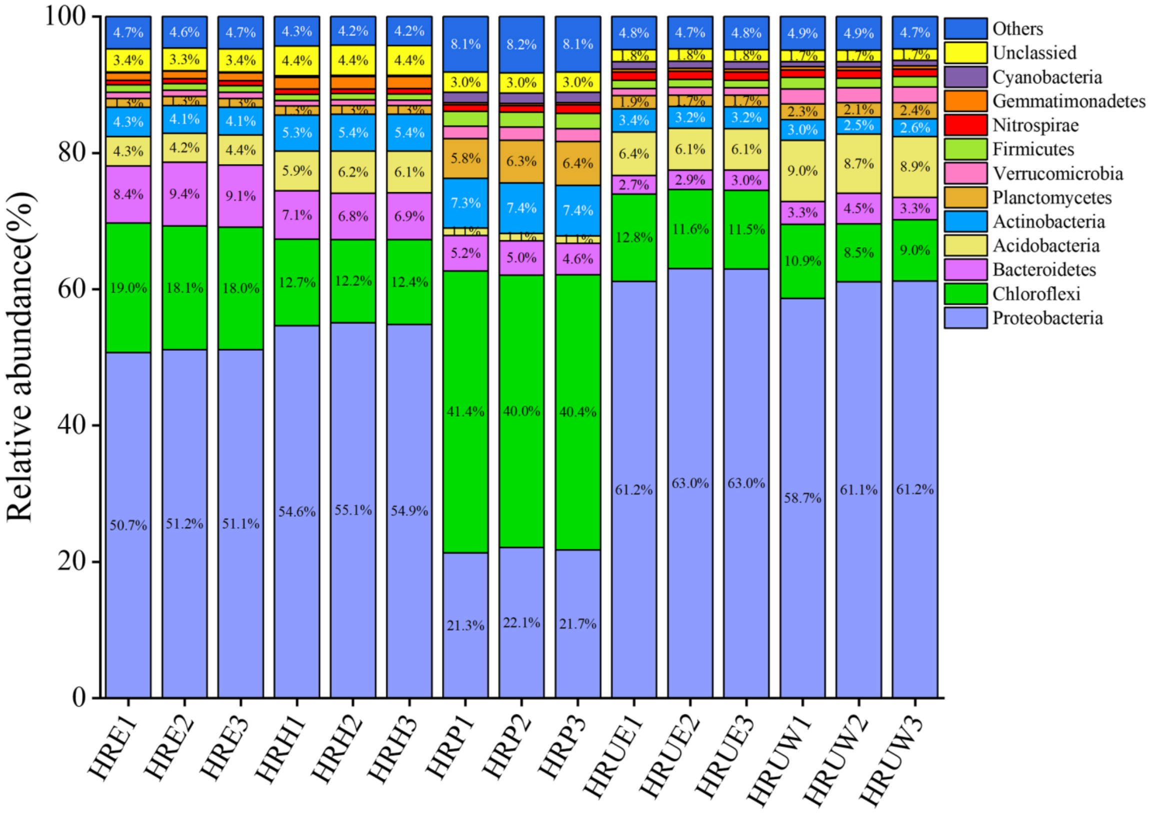

Figure S1. Composition of bacteria communities in fifteen sediment samples with relative abundance at phylum level.
